# New insights into pterosaur cranial anatomy: X-ray imaging reveals palatal structure and evolutionary trends

**DOI:** 10.1038/s42003-024-06132-6

**Published:** 2024-04-12

**Authors:** He Chen, Shunxing Jiang, Alexander W. A. Kellner, Xiaolin Wang

**Affiliations:** 1 School of Ecology, Sun Yat-sen University, Shenzhen, 510006 China; 2 Key Laboratory of Vertebrate Evolution and Human Origins, Institute of Vertebrate Paleontology and Paleoanthropology, Chinese Academy of Sciences, Beijing, 100044 China; 3 University of Chinese Academy of Sciences, Beijing, 100049 China; 4 Laboratory of Systematics and Taphonomy of Fossil Vertebrates, Department of Geology and Paleontology, Museu Nacional/UFRJ, Rio de Janeiro, 20940-040 Brazil

**Keywords:** Palaeontology, Herpetology

## Abstract

Among the least studied portion of the pterosaur skeleton is the palate, which tends to be poorly preserved and commonly only visible from one side (the ventral portion). Even in well-preserved specimens, the bones tend to be fused, with the limits of individual palatal elements obscured. To shed new light on this region, we employed advanced X-ray imaging techniques on the non-pterodactyloid *Kunpengopterus* (Wukongopteridae), and the pterodactyloids *Dsungaripterus* (Dsungaripteridae), *Hongshanopterus* (Istiodactylidae), and *Hamipterus* (Hamipteridae). Our analyses revealed the presence of sutures between palatal bones in *Dsungaripterus and Kunpengopterus*, which resulted in different interpretations of the relation between palatine, ectopterygoid, and pterygoid, leading to a new identification of the palatal openings. Furthermore, our study shows six main observations such as the variation of the angle between the palatine rami and the variation in the relative sizes of the palatal openings. We also point out that the presence of a maxillopalatine fenestra (previously identified as postpalatine fenestra), is unique within Diapsida. Although much more work needs to be done, we showed that advanced X-ray imaging techniques open a window for understanding pterosaur cranial anatomy and provide a new perspective for investigating the evolutionary history of these flying reptiles.

## Abstract (web summary)

A study on the palatal region of pterosaurs suggests different bone interpretations and new identification of the palatal openings, pointing out that the maxillopalatine fenestra in pterosaurs is unique within Diapsida.

## Introduction

Pterosaurs, the first vertebrates to achieve powered flight, have been a subject of fascination for scientists for decades. Identifying individual bones in the skull of extinct clades can be challenging given the lack of living descendants for comparison. This challenge is particularly challenging for pterosaurs, a group of extinct flying reptiles that lived during the Mesozoic Era. However, an accurate interpretation of their anatomy is the fundamental prerequisite for a wide range of studies, including phylogeny, ecology, biology, and functional morphology^1–9^.

As generally known the normal condition of preservation regarding pterosaurs is that of crushed or flattened specimens and three-dimensionally preserved material is exceedingly rare (e.g.,^10,11^). Additionally, cranial bones, including the palate, often exhibit fusion. Consequently, gaining an understanding of detailed skull architecture, especially the conformation of the palate in pterosaurs, has always been difficult. Furthermore, the identification of the limits of several elements generally still lacks consensus^12–14^.

Questions regarding the construction of the pterosaur palate have been the subject of debate since the 19th century^15–22^. While several new specimens have come to light over the years^10,23–37^, the lack of clarity regarding the boundaries between different palatal bones has resulted in an ongoing debate^38–45^. Recently, a specimen of the Jurassic pterosaur *Dorygnathus banthensis*, featuring several unfused cranial bones, presented a very different configuration of the palatal portion of the maxilla^13^. This discovery led to a reinterpretation of the palate^13^ with broad implications (e.g., refs. ^14,38,41^). However, controversy persists, particularly concerning the extent and shape of the palatines and whether a single, generalized palatal pattern configuration can be applied to Pterodactyloidea^23,38,40–43^.

Here we review the palate structure in various pterosaur taxa (Table 1), including both pterodactyloids and non-pterodactyloids. We introduce new insights based on CT-scans and CL-scans of *Kunpengopterus* (Wukongopteridae), *Hongshanopterus* (Istiodactylidae), *Hamipterus* (Hamipteridae), and *Dsungaripterus* (Dsungaripteridae). Our findings shed light on the boundaries of several palatal bones and propose a new configuration for the palate of numerous pterodactyloid clades. Additionally, we reinterpret distinct configurations for the palate of several non-pterodactyloid clades, offering insights into potential evolutionary trends of this region in pterosaurs.

**Table 1:** Specimens with palatal information

Specimens	Taxon	Institutional abbreviations
SMNS 18969 (50,184 + 50,914 + 51,827)	*Dorygnathus banthensis*	Staatliches Museum fur Naturkunde, Stuttgart, Germany
CM 11,424	*Campylognathoides liasicus*	Carnegie Museum Pittsburgh, USA
GSM 3166	*Parapsicephalus purdoni*	British Geological Survey, Nottingham, UK
IGO-V 208	*Cacibupteryx caribensis*	Museo Mario Sánchez Roig, Instituto de Geología y Paleontología, La Habana, Cuba
NHM R 2786 (Nr.55)	*Rhamphorhynchus muensteri*	Natural History Museum, London, UK
CM 11434	*Rhamphorhynchus muensteri*	Carnegie Museum Pittsburgh, USA
GPIB 1304 (Nr. 109)	*Scaphognathus crassirostris*	Geologische Institut fur Palaontologie der Universitat Bonn, Germany
NMS G.2021.6. 2	*Dearc sgiathanach*	National Museums Scotland, Edinburgh, UK
^a^IVPP V 23674	*Kunpengopterus sinensis*	Institute of Vertebrate Paleontology and Palaeoantropology, Beijing, China
BSP 1936 I 50	*Aurorazhdarcho micronyx*	Staatliche Naturwissenschaftliche Sammlungen Bayerns/Bayerische Staatssammlung fur Paläontologie und Geologie, Munich, Germany
^a^IVPP V 14852	*Hongshanopterus lacustris*	Institute of Vertebrate Paleontology and Palaeoantropology, Beijing, China
IVPP V 18943.1, ^a^IVPP V 18943.3	*Hamipterus tianshanensis*	Institute of Vertebrate Paleontology and Palaeoantropology, Beijing, China
RGM 401 880	*Anhanguera spielbergi*	National Natuurhistorisch Museum/Naturalis, Leiden, The Netherlands
AMNH 25555	*Anhanguera santanae*	American Museum of Natural History, New York, USA
AMNH 22555	*Anhanguera santanae*	American Museum of Natural History, New York, USA
SNSB-BSPG 1982 I 90	*Anhanguera santanae*	Staatliche Naturwissenschaftliche Sammlungen Bayerns/Bayerische Staatssammlung fur Paläontologie und Geologie, Munich, Germany
SNSB-BSPG 1982 I 89	*Anhanguera araripensis*	Staatliche Naturwissenschaftliche Sammlungen Bayerns/Bayerische Staatssammlung fur Paläontologie und Geologie, Munich, Germany
SAO 16494	*Anhanguera araripensis*	Sammlung Oberli, St. Gallen, Switzerland
MN 4805-V	*Anhanguera blittersdorffi*	Museu Nacional/Universidade Federal do Rio de Janeiro, Rio de Janeiro, Brazil
BSP 1987 I 46	*Tropeognathus mesembrinus*	Staatliche Naturwissenschaftliche Sammlungen Bayerns/Bayerische Staatssammlung fur Paläontologie und Geologie, Munich, Germany
PTH 1951.84	*Gnathosaurus subulatus*	Philosophische-Theologische Hochschule, Eichstätt, Germany
PMOL-AP00031	*Liaodactylus primus*	Paleontological Museum of Liaoning, Shenyang Normal University, Shenyang, China
^a^IVPP V 4063, 26257, 26258	*Dsungaripterus weii*	Institute of Vertebrate Paleontology and Palaeoantropology, Beijing, China
MN 4726-V	*Caupedactylus ybaka*	Museu Nacional/Universidade Federal do Rio de Janeiro, Rio de Janeiro, Brazil
IMCF 1052	*Tupuxuara leonardii*	Iwaki Coal and Fossil Museum, Iwaki, Japan
MNHN 1908-24^31^	*Pteranodon*	Museum National d’Histoire Naturelle, Paleontologie, Paris, France
No.2594^64^	*Pteranodon ingens*	Peabody Museum of Yale University, USA
FMNH 25026	*Nyctosaurus gracilis*	Field Museum of Natural History, Chicago, USA

^a^The specimens have been CT-scanned or CL-scanned in this study.

## Materials and methods

The objective of this study was to examine the morphology and internal structure of palatal bones using X-ray micro-computed tomography and laminography. The specimens were scanned using the following different types of X-ray instruments, depending on their different preservation and size.

**Fig. 1:**
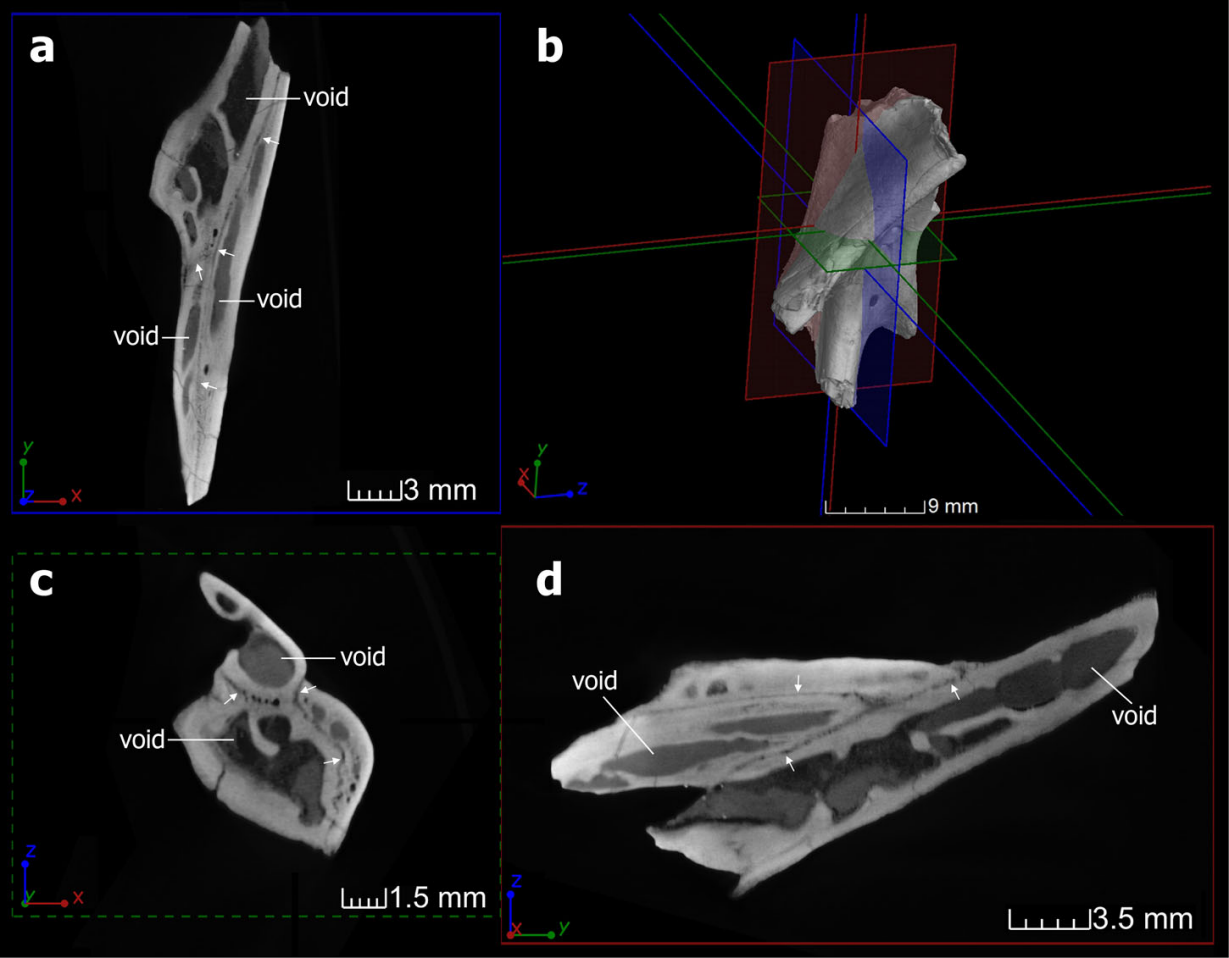
CT slices of *Dsungaripterus weii* IVPP V 26257. **a**, **c**, **d** CT slices, showing the sutures pointed by arrows and internal structure. **b** Shows the place where those CT slices are. This specimen’s CT-rendered result is shown in Fig. 2c.

X-ray micro-computed tomography (CT): The specimen of *Dsungaripterus* (IVPP V 4063) was scanned using a GE v|tome|x m dual tube 300/180 kV system in the Key Laboratory of Vertebrate Evolution and Human Origins, Institute of Vertebrate Paleontology and Paleoanthropology (IVPP), Chinese Academy of Sciences (CAS). The specimen was scanned with a beam energy of 240 kV and a flux of 200 μA at a resolution of 35.88 μm per pixel using a 360° rotation with a step size of 0.18°. Two specimens of *Dsungaripterus* (IVPP V 26257, IVPP V 26258) and one specimen of *Hamipterus* (IVPP V 18943.3) were scanned using the 225 kV micro-computerized tomography in IVPP (developed by the Institute of High Energy Physics, CAS). The specimens of *Dsungaripterus* and *Hamipterus* were scanned with a beam energy of 130 kV and a flux of 150 μA at a resolution of 34.50 μm per pixel, and 120 kV and a flux of 120 μA at a resolution of 25.09 μm per pixel respectively, using a 360° rotation with a step size of 0.5°. A total of 720 projections were reconstructed in a 2048 × 2048 matrix of 1536 slices using a two-dimensional reconstruction software (IVPP-IHEP) developed by the Institute of High Energy Physics, CAS. Figure 1 shows examples of the scan results of the IVPP V 26257, which are primary data on which the palatal bones were reconstructed.

X-ray micro-computed laminography (CL): The flattened specimens of *Kunpengopterus* and *Hongshanopterus* were scanned using the CL scanner in the lab at the IVPP (developed by the Institute of High Energy Physics, CAS for flat specimens). The specimens were scanned with a beam energy of 80 kV and a flux of 60 μA at a resolution of 9.02 μm per pixel, and 70 kV and a flux of 70 μA at a resolution of 33.7 μm per pixel, respectively, using a 360° rotation with a step size of 0.5°. A total of 360 image slices with a size of 2048 by 2048 were reconstructed using a modified Feldkamp algorithm developed by the Institute of High Energy Physics, CAS.

The imagery data obtained from CT and CL were using VG Studio Max 3.0 (Volume Graphics, Heidelberg, Germany) to segment, render, and reconstruct the 3D bones (Supplementary Data 1), and make the video 1.

Regarding pterosaur phylogenies, there are several proposals/hypothesis published, with two main phylogenetic schemes (Kellner^2^ and Unwin^3^), that have been modified with the addition of new taxa and the employment of different methodologies (e.g., refs. ^4,9,46^). Here we have followed mainly Kellner et al.^47^.

### Reporting summary

Further information on research design is available in the Nature Portfolio Reporting Summary linked to this article.

## Results and discussion

**Fig. 2:**
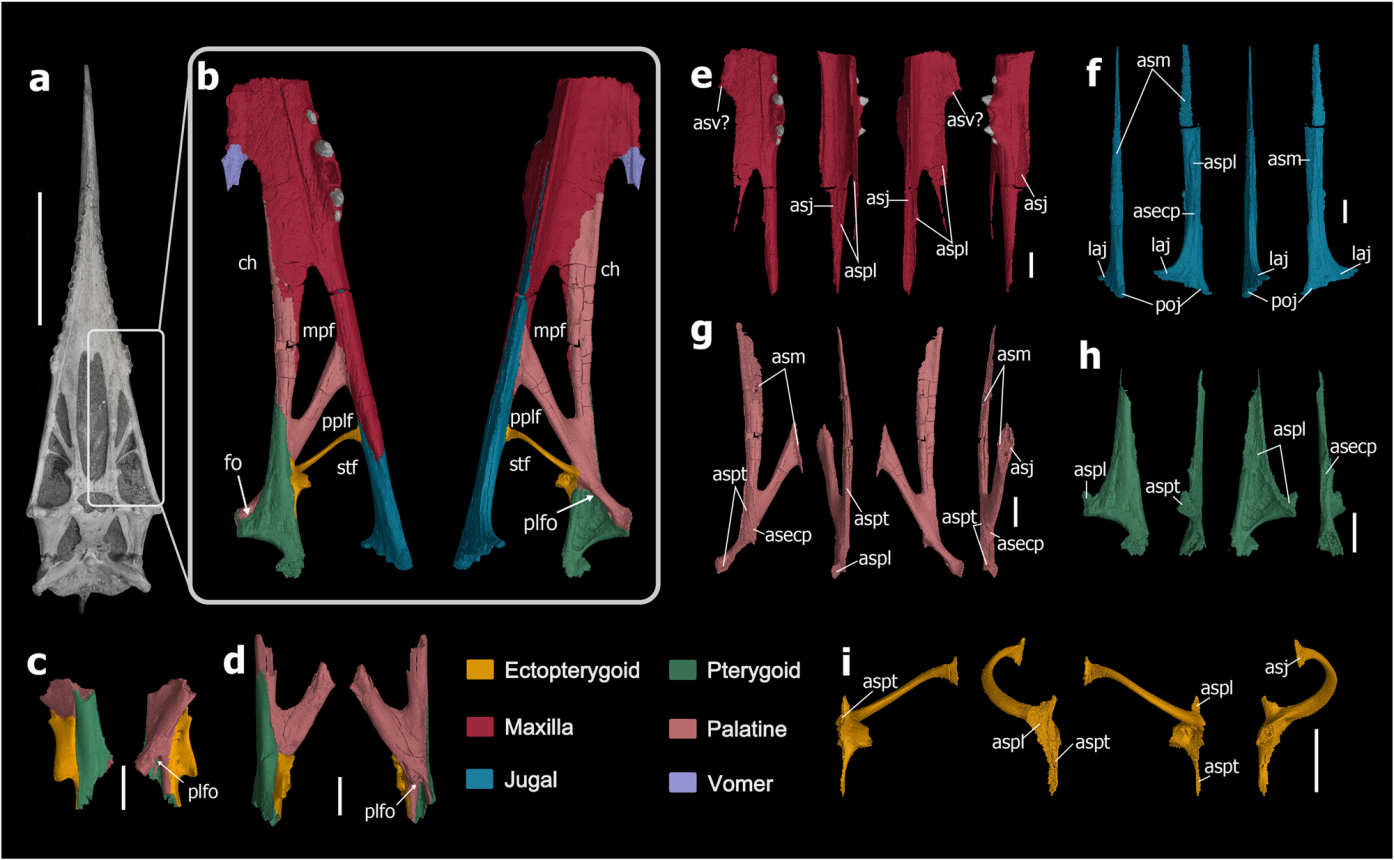
The three specimens of *Dsungaripterus weii* and their CT-rendered results. **a** The skull IVPP V 4063 in ventral view. **b** CT-rendered the left part of the palate of IVPP V 4063 in ventral and dorsal views. **c** CT-rendered model of IVPP V 26258, showing the contact region of the right side of the palatine, pterygoid, and ectopterygoid in ventral and dorsal views. **d** CT-rendered model of IVPP V 26257 showing the contact region of the left side of the palatine, pterygoid, and ectopterygoid, in ventral and dorsal views. **e** The posterior part of the maxilla in ventral, medial, dorsal, and lateral views. **f** The maxilla process of the jugal in ventral, medial, dorsal, and lateral views. **g** The palatine in ventral, medial, dorsal, and lateral views. **h** The pterygoid with unclear posterior boundary in ventral, medial, dorsal, and lateral views. **i** the ectopterygoid in ventral, medial, dorsal, and lateral views. Abbreviations: asecp articular surface of ectopterygoid, asj articular surface of jugal, asm articular surface of maxilla, aspl articular surface of palatine, aspt articular surface of pterygoid, asv articular surface of vomer, ch choana, fo foramen, laj lacrimal process of jugal, mpf maxillopalatine fenestra, plfo palatine foramen, pplf postpalatine fenestra, poj postorbital process of jugal, stf subtemporal fenestra. Scale bars: **a** = 100 mm; **c**–**i** = 10 mm.

**Fig. 3:**
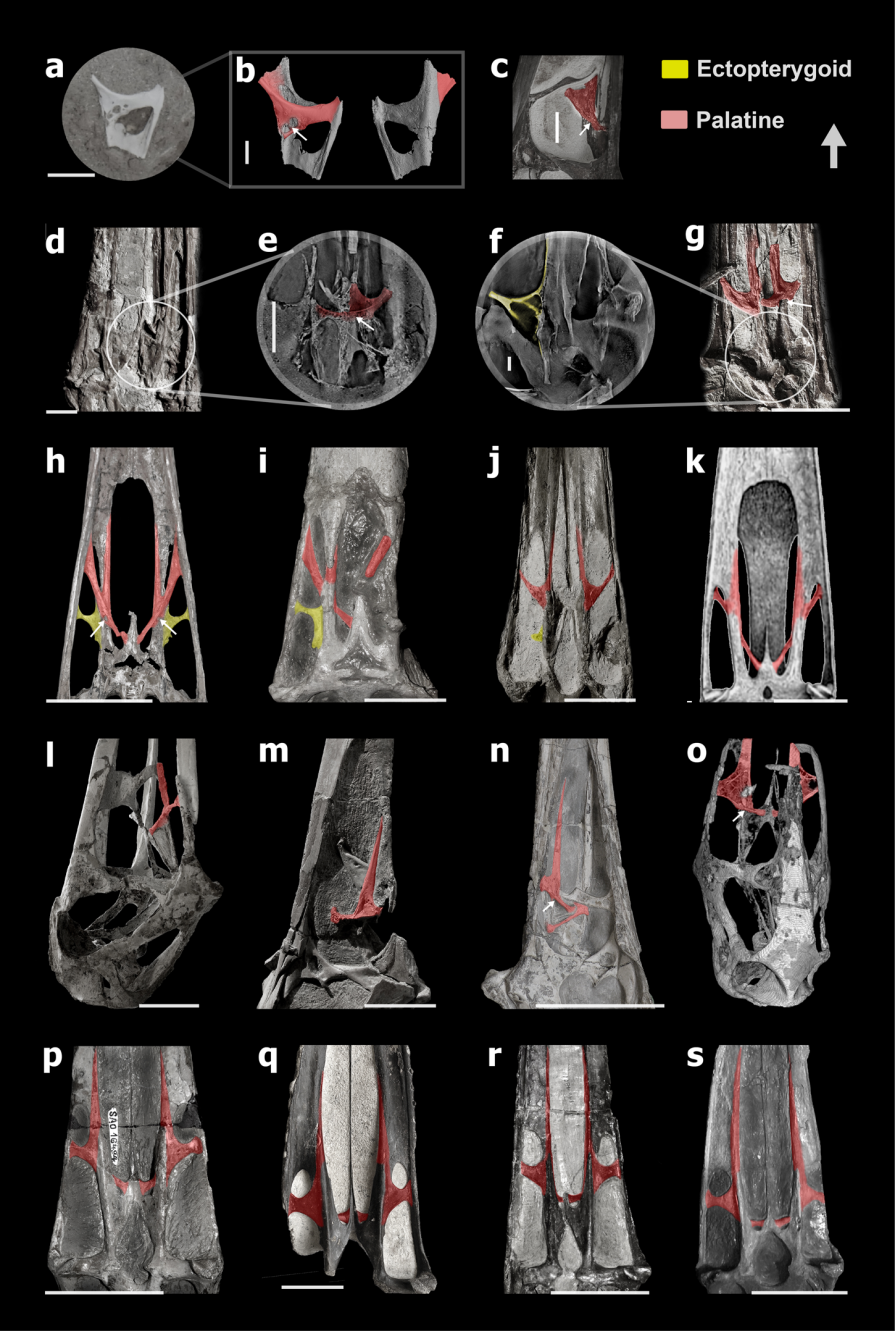
The similar structure on the palatal region in pterodactyloids. **a** A piece of skull of *Hamipterus tianshanensis* (left side) IVPP V 18943.3. **b** CT-rendered bone corresponding to the (**a**) in dorsal and ventral view. **c** A close-up shot of the palatine of *Ludodactylus* SMNK PAL 3828 in dorsal view. **d** the posterior part of the palate of *Hongshanopterus lacustris* IVPP V 14852 in ventral view, modified from ref. ^35^. **e** CL-scan results corresponding to the circle area in (**d**). **f** CL-scan results corresponding to the circle area in (**g**). **g** The posterior part of palate of *Kunpengopterus sinensis* IVPP V 23674 in dorsal view, modified from ref. ^41^. **h** The posterior part of the palate of *Caupedactylus ybaka* MN 4726-V in dorsal view, modified from ref. ^38^. **i** The posterior part of the palate of *Tupuxuara leonardii* IMCF 1052. **j** The posterior part of the palate of *Gnathosaurus subulatus* SOS 4580 (cast) in ventral view. **k** The posterior part of the palate of *Pteranodon*, modified from ref. ^64^. **l**–**r** Seven skulls of *Anhanguera* focus on the palate region, and they are RGM 401880 (lateral-dorsal view on the right side, photographed by Dr. T. Rodrigues), AMNH 22555 (lateral view on the right side), SNSB-BSPG 1982 I 89 (lateral view on the left side), AMNH 25555 (dorsal-lateral view on left side, CT-rendered model modified from ref. ^33^, SAO 16494 (ventral view, photographed by Dr. T. Rodrigues), SNSB-BSPG 1982 I 89 (ventral view, photographed by Dr. T. Rodrigues), MN 4805-V (ventral view), respectively. **s** The posterior part of palate of *Tropeognathus mesembrinus* BSP 1987 I 46 (cast) in ventral view. Scale bar: **a**, **d**, **e**, **g** = 10 mm; **b** = 4.2 mm; **j** = 20 mm; **f** = 1 mm; **h**, **i**, **k**, **l**, **m**, **p**, **q**, **r** = 50 mm; **n** = 100 mm; **s** = 85 mm.

The utilization of X-ray techniques (including CT-scan and CL-scan) has enabled us to investigate the internal structure of the palate of several pterosaurs, thus revealing the boundaries of several bones despite their strongly fused external surface. The CT-scans of the thick palatal elements in all three *Dsungaripterus* specimens analyzed (e.g., Fig. 1) show irregular edges of the sutures between the bones, resembling the crenelate boundary of postage stamps (e.g., Fig. 1), which differentiate them from cracks or fractures. In the case of the most complete specimen of *Dsungaripterus* (IVPP V 4063), most sutures are clearly identifiable, allowing for the separation of the bones (Fig. 2). However, the CT-scans of the specimen of *Hamipterus* (IVPP V 18943.3) (Fig. 3a, b) studied here did not reveal any sutures, suggesting that all palatal elements are fused.

**Fig. 4:**
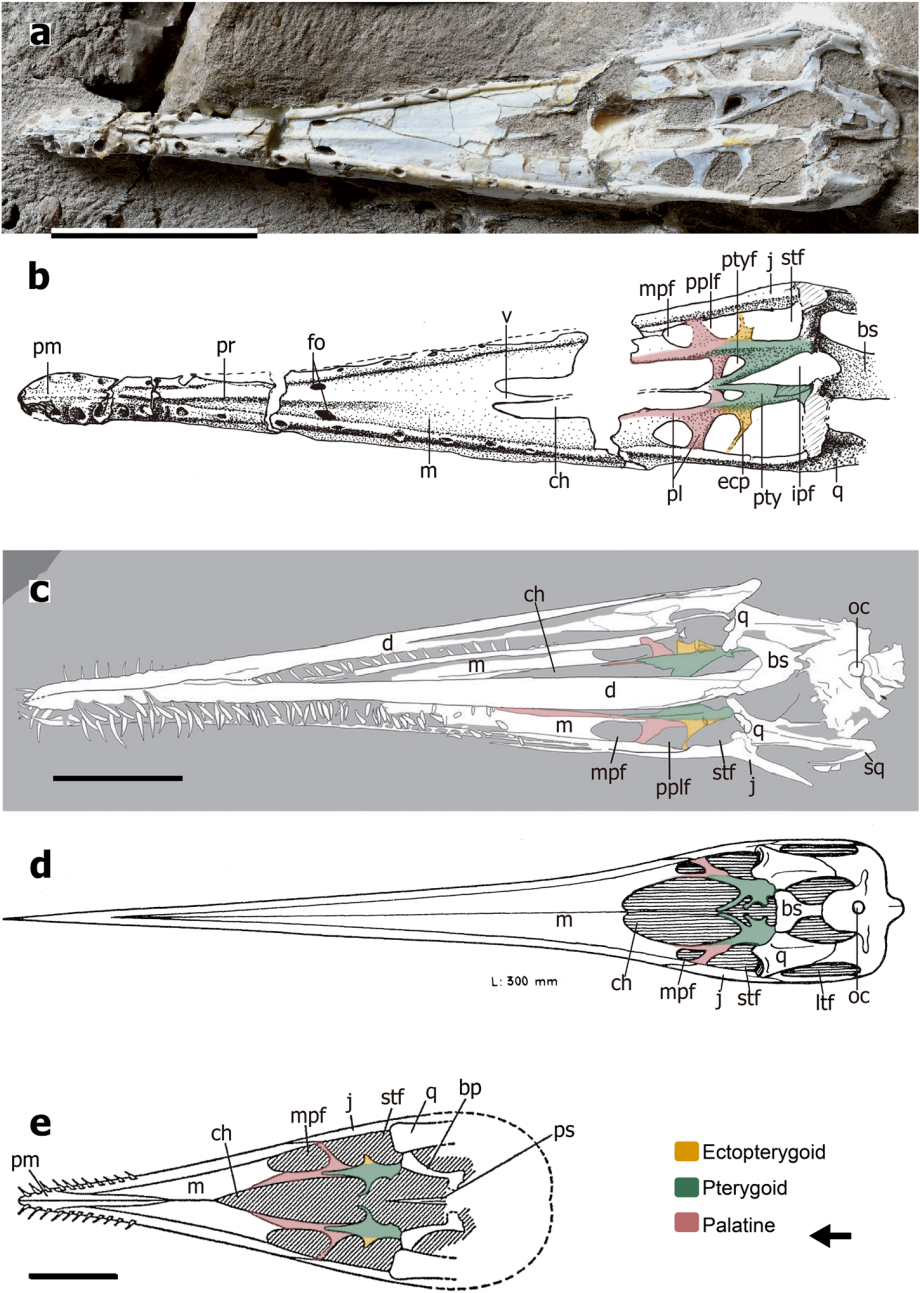
The nearly complete palatal region from the other four pterodactyloids. **a** Photo of a skull of *Hamipterus tianshanensis* IVPP V 18943.1 seen on the ventral side, and **b** is the sketch drawing of it. **c** Line drawings of the ventral side of *Liaodactylus primus* PMOL-AP00031, modified from ref. ^42^. **d** Reconstruction of the ventral side of *Nyctosaurus gracilis* FM P 25026, modified from ref. ^12^. **e** Reconstruction of the ventral side of *Aurorazhdarcho micronyx* BSP 1936 I 50, modified from ref. ^23^. Abbreviations: bp basipterygoid, bs basisphenoid, ch choana, d dentary, ecp ectopterygoid, fo foramen, ipf interpterygoid fenestra, j jugal, ltf lower temporal fenestra, m maxilla, mpf maxillopalatine fenestra, oc occipital condyle, pm premaxilla, pl palatine, pplf postpalatine fenestra, pr palatal ridge, pty pterygoid, ptyf pterygoid fenestra, ps parasphenoid, q quadrate, sq squamosal, stf subtemporal fenestra, v vomer. Scale bars: **a**, **b** = 50 mm, **c** = 20 mm, **e** = 5 mm.

In the case of the compressed specimens of *Hongshanopterus* and *Kunpengopterus*, we use CL-scan to identify any potential preserved suture. Regarding *Hongshanopterus* (Fig. 3d, e), due to the preservation that shows several fractures, no sutures could be confidently identified. In the case of *Kunpengopterus* (Fig. 3f, g), there are fewer fractures, and the boundary between the ectopterygoid and pterygoid could be seen (Fig. 3f). The remaining palatal elements in this specimen appeared to be fused. Despite the fact that no clear sutures were found in the specimens from *Hamipterus* and *Hongshanopterus* studied here (Figs. 3a, b, 4a, b), and only one suture was observed in *Kunpengopterus* (Fig. 3f, g), CT-scan and CL-scan proved to be very informative since they made it possible to observe the dorsal side of the palate.

## Palatal reconstruction of *Dsungaripterus weii* (Dsungaripteridae)

**Fig. 5:**
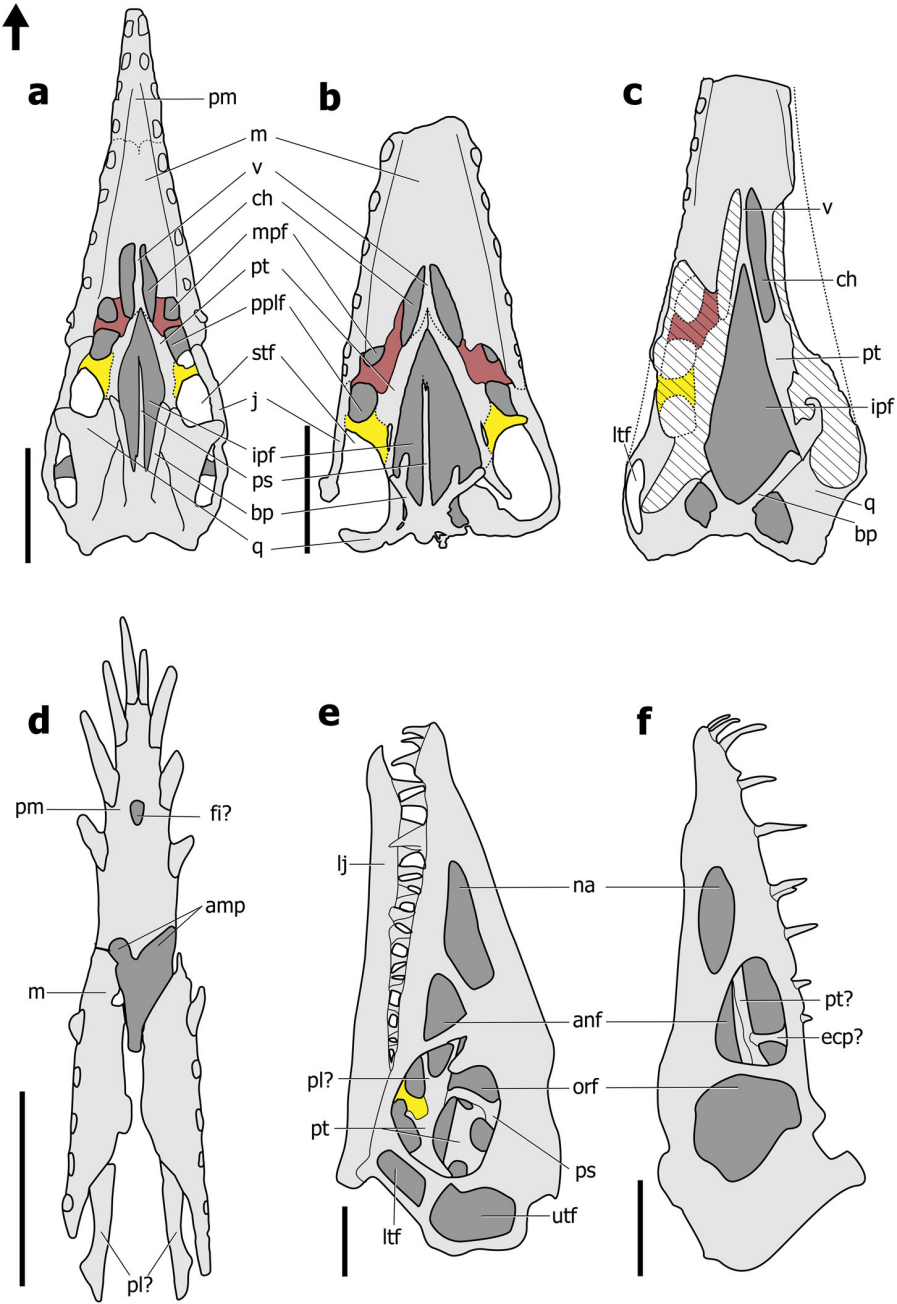
Line drawings of the palatal region in non-pterodactyloid. **a** *Rhamphorhynchus muensteri* CM 11434, modified from ref. ^33^. **b** *Cacibupteryx caribensis* IGO-V 208, modified from ref. ^36^. **c** *Parapsicephalus purdoni* GSM 3166, modified from ref. ^16^, and the skull length is about 140 mm. **d** *Dorygnathus banthensis* SMNS 18969, modified from ref. ^13^. **e** *Campylognathoides liasicus* CM 11424, modified from ref. ^49^. **f** *Scaphognathus crassirostris* GPIB 1304 (Nr. 109), modified from ref. ^25^. The red represents the palatine and the yellow represents the ectopterygoid. Abbreviations: amp apertura maxillo-premaxillaris, anf antorbital fenestra, bp basipterygoid, ch choana, ecp ectopterygoid, fi foramen incisivum, ipf interpterygoid fenestra, j jugal, lj lower jaw, ltf lower temporal fenestra, m maxilla, mpf maxillopalatine fenestra, na naris, orf orbital fossa, pl palatine, pm premaxilla, pplf postpalatine fenestra, ps parasphenoid, pt pterygoid, q quadrate, stf subtemporal fenestra, utf upper temporal fenestra, v vomer. Scale bar: **a**, **f** = 20 mm; **b**, **d** = 50 mm; **e** = 10 mm.

The CT-scans of IVPP V 4063 provide a relatively clear picture of the anatomy structure of the maxilla, palatine, pterygoid, and ectopterygoid, which differs from previous work on *Dsungaripterus*^43^. Except for the contact with the vomer, the boundary of the posteroventral side of the maxilla is well established (Fig. 2a, b, e). The jugal process of the maxilla contacts the jugal (Fig. 2f) ventrally, forming the lateral edge of the palate, and extends posteriorly to the ectopterygoid (Video 1 and Fig. 2b). Compared to the maxilla of *Dorygnathus banthensis*^13^ (Fig. 5d), the maxilla of *Dsungaripterus* presents a process on the ventromedial side directed posteriorly that is here referred to as the palatine process of the maxilla (Video 1 and Fig. 2e). This new process contacts the anterior ramus of the palatine dorsomedially (Video 1 and Fig. 2b, e). With this new interpretation, the most anterior fenestra positioned lateral to the choanae is bordered by the maxilla anteriorly and the palatine posteriorly, and therefore renamed here as the maxillopalatine fenestra. This opening has been called by most authors as the postpalatine fenestra (e.g., refs. ^12,31,38^) or suborbital fenestra^13^. CT-scans did not reveal any suture at the grooves preserved on the ventral side of the maxilla that has been previously interpreted as the boundaries of the maxilla and the palatine in several pterodactyloid taxa (e.g., refs. ^28,38,43,48^).

The 3D reconstruction of the palatine in *Dsungaripterus* shows two dorsoventrally flat palatine rami that form a distinctive y-shape (Video 1 and Fig. 2b–d, g). According to our new interpretation, the palatine contacts all other palatal bones except for the ventral portion of the premaxilla (no information is presently available for the vomer) and is involved in almost all palatal openings, except for the interpterygoid fenestra. The anterior ramus of the palatine ventromedially contacts the palatine process of the maxilla, and both separate the maxillopalatine fenestra medially from the choana. The lateral ramus of the palatine contacts the lateral edge of the palate, separating the maxillopalatine fenestra from the postpalatine fenestra referred to by others as the infraobital vacuity (e.g., refs. ^18,36^), the pterygoid-ectopterygoid fenestra^13,14^, or the secondary subtemporal fenestra^37,40,42^. The lateral ramus of the palatine is elongated and overlays the anterior ramus on the dorsal side at the posterior region (Video 1 and Fig. 2b–d, g). Medially, this lateral ramus becomes thinner, and contacts the dorsal surface of the pterygoid. At the posterior end, it contacts the opposing palatine, and both form the posterior margin of the choanae. There is a small posterior process that contacts the pterygoid dorsally and the ectopterygoid laterally and contacts the subtemporal fenestra (Video 1 and Fig. 2b–d, g). A small foramen perforates the lateral rami of the palatine at the contact surface with the pterygoid and is here called the palatine foramen (Video 1 and Fig. 2b–d). Since this foramen is ventrally covered by the pterygoid, it can only be observed from the posterodorsal view.

The pterygoid is a plate-like bone with two processes (Video 1 and Fig. 2b, h). The anterior process gradually tapers anteriorly, and it ventrally contacts the palatine anterior ramus. The medial process of the pterygoid covers ventrally the posterior part of the palatine (Video 1 and Fig. 2b, h), and medially contacts its counterpart, separating the choanae and the interpterygoid fenestra. Posterior to the choanae, the pterygoid foramen is observed between the palatine and the pterygoid (Fig. 2b). The articulation surface of the pterygoid with the ectopterygoid shows a rough texture (Video 1 and Fig. 2b, h). In the present specimen (IVPP 4063), the pterygoid fuses with the quadrate and probably also with the basisphenoid. The medial process of the pterygoid could have been anteriorly in contact with the vomer, as the slender vomer expands posteriorly to the posterior margin of the choanae^43^.

Previously, the ectopterygoid was described as the “lateral process of the pterygoid”^43^. This bone can be divided into two parts (Video 1 and Fig. 2b, i). The medial part is curved dorsoventrally, flattened, and has the dorsal surface perforated by several foramina (Video 1 and Fig. 2b, i). Medially, it contacts the palatine and the pterygoid. The lateral portion of the ectopterygoid is positioned anterolaterally relative to the medial part, and it is a slender, tubular, and parabolic arc-shaped element, that laterally contacts the jugal (Fig. 2f). The ectopterygoid separates the postpalatine fenestra from the subtemporal fenestra (Video 1 and Fig. 2b).

## The palate of *Kunpengopterus sinensis* (Wukongopteridae)

**Fig. 6:**
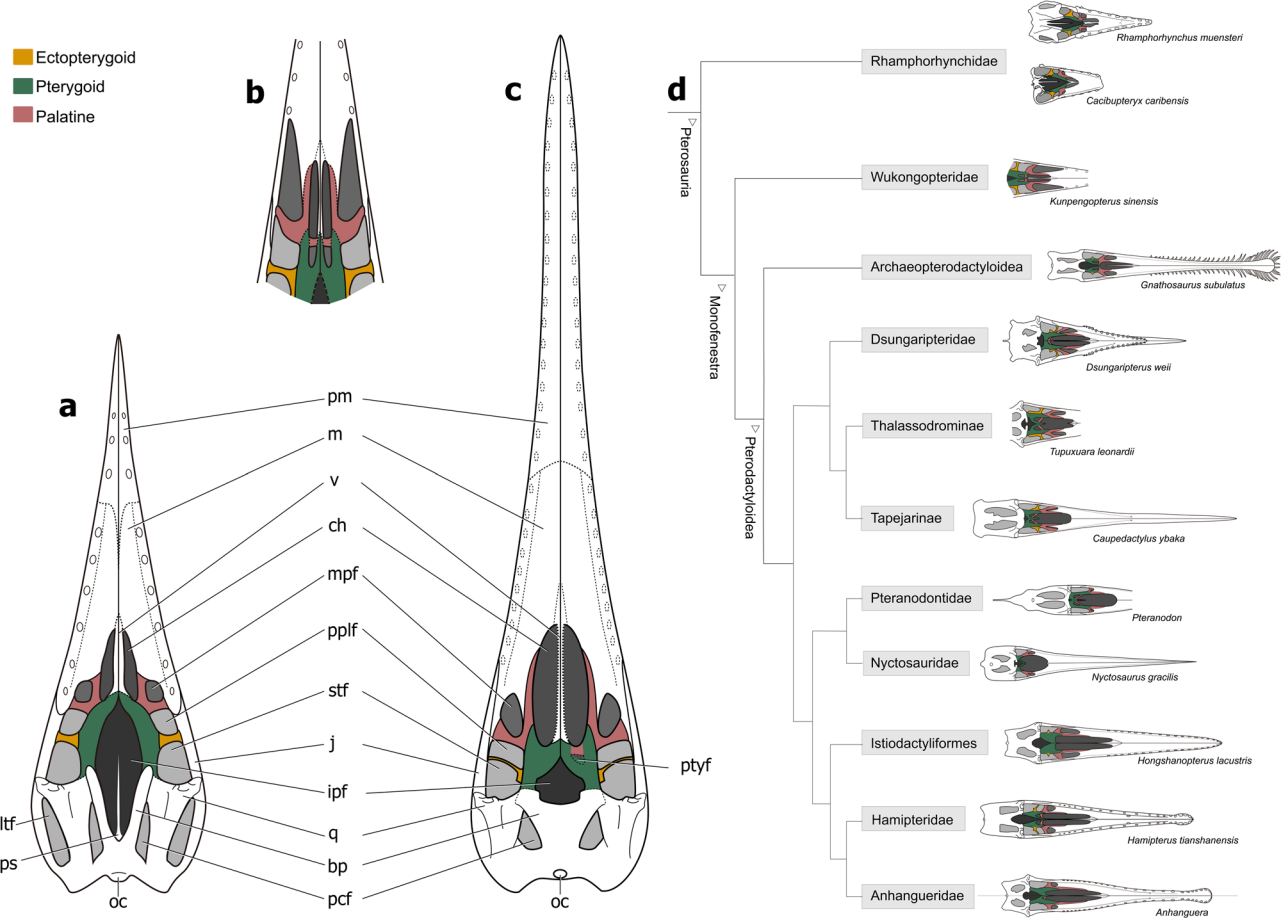
The line drawings of three hypothetical models of the palate in ventral view and its evolution in pterosaurs. **a** palate model based on *Rhamphorhynchus* and *Cacibupteryx*. **b** Palate model of *Kunpengopterus*. **c** Palate model of pterodactyloids. **d** The cladogram was simplified and modified from ref. ^47^. Line drawings of the palates of *Rhamphorhynchus*, *Cacibupteryx*, *Gnathosaurus*, *Caupedactylus*, *Pteranodon*, *Nyctosaurus*, *Hongshanopterus*, and *Anhanguera* were reconstructed and modified from previous publications^12,27,33,35,36,38,64^. Abbreviations: bp basipterygoid, ch choana, ipf interpterygoid fenestra, j jugal, ltf lower temporal fenestra, m maxilla, mpf maxillopalatine fenestra, oc occipital condyle, pcf posterior cranial fenestra, pm premaxilla, pplf postpalatine fenestra, ps parasphenoid, ptyf pterygoid fenestra, q quadrate, stf subtemporal fenestra, v vomer.

The specimen of *Kunpengopterus sinensis* (IVPP 23674) also preserves a similar “y” shape palatine and also shows a palatine foramen which, as in *Dsungaripterus*, can only be seen from the dorsal side (Fig. 3g). Based on the CL-scans, the bone previously described as the “lateral process of the pterygoid”^41^ is the ectopterygoid that shows a clear boundary with the pterygoid (Fig. 3f). There are three palatal openings lateral to the choana, similar to that observed in *Dsungaripterus*. Furthermore, the palatine and ectopterygoid are also similar in both taxa, suggesting that both have developed a similar palatal bone pattern. (Fig. 6b). The main difference is the presence of a small pterygoid foramen in *Dsungaripterus*, while *Kunpengopterus* exhibits a pterygoid fenestra that is more similar to the one observed in *Anhanguera*^27^ (Fig. 3), *Hamipterus*^48^ (Fig. 4), and *Caupedactylus*^38^ (Fig. 3h).

## The palate in non-pterodactyloids

There are not many specimens of non-pterodactyloid pterosaurs that allow the observation of the palate^13,16,24,25,40,44,49,50^ (Table 1). Among the best-preserved materials are BML R 2786^18^ and CM 11434^33^ (Fig. 5a) representing *Rhamphorhynchus*, and *Cacibupteryx* (IGO-V 208^36^ Fig. 5b), two Jurassic rhamphorhynchids. Our basic reconstruction follows the one published by Ösi et al.^13^, but with different interpretations of bones and palatal openings (Fig. 6a). According to our interpretation, the palatine has two rami, which form the medial and posterior margin of the maxillopalatine fenestra. Therefore, the opening identified by Ösi et al.^13^ as the suborbital fenestra and pterygoectopterygoid fenestra should be redesignated as the maxillopalatine and postpalatine fenestrae. Furthermore, Ösi et al.^13^ have identified the palatine as being a long, flattened element positioned lateral to the choanae. If, indeed, Ösi et al.^13^ are correct, then the palatal configuration of *Dorygnathus*, which also shows an apertura maxillo-premaxillaris not recognized in other non-pterodactyloids, might differ from what we present here.

Another member of the Rhamphorhynchidae with a well-preserved palate that, albeit still preserved in the matrix, was observed through CT-scan^51^ is *Dearc sgiathanach*. Based on our analysis of the CL-scan of *Kunpengopterus*, the “y” shaped element observed on the dorsal part that was interpreted as being the ectopterygoid^51^ is here regarded to be the palatine. This bone is positioned posterior to the margin of the most anterior opening -the maxillopalatine fenestra-. Both palatines meet at the midline and contact the vomer. The original openings designated as the suborbital fenestra and pneumatic foramen (perhaps they meant the pterygoid-ectopterygoid fenestra) are, according to our interpretations, the maxillopalatine fenestra and the postpalatine fenestra.

There are at least two other quite distinctive palate configurations present in non-pterodactyloids. The most extreme, as has been already pointed out before, is found in anurognathids where most elements are reduced to rodlike structures^32,50,52–54^. The other distinctive palate has been reported in the wukongopterid *Kunpengopterus* with different relation of the palatal openings, particularly the huge size of the postpalatine fenestra^41^. Based on our study, what originally has been identified as the postpalatine and the secondary subtemporal fenestrae are actually the maxillopalatine and the postpalatine fenestrae, respectively (Fig. 6b). Furthermore, we here reinterpret the ectopterygoid and the lateral process of the pterygoid of the original description^41^ as the palatine and the ectopterygoid, respectively (Fig. 6b).

Previous reconstructions of the palate of other non-pterodactyloid taxa are also reinterpreted here. The sole known specimen of *Parapsicephalus* (GSM 3166)^16,44^ is not very well preserved and shows only the partial right side of the palate that might follow the same pattern (Fig. 5c) as we report for the rhamporhynchids discussed previously. In *Campylognathoides* (CM 11424)^24,49^ the bone observable through the orbit and identified as the ectopterygoid^24^ is here the same as our interpretation (Fig. 5e). Lastly, in the palate of *Scaphognathus* (GPIB 1304)^25,40^ the bones identified as the ectopterygoid might be the palatine (Fig. 5f).

## The palate in pterodactyloids

The new interpretation of the palatal structure in *Dsungaripterus* (Dsungaripteridae) can also be applied to other pterodactyloid clades. CT-scans and CL-scans of *Hongshanopterus*^35^ (Istiodactylidae) (Fig. 3d, e) and *Hamipterus*^48^ (Hamipteridae) allowed the examination of the dorsal side of the palate, indicating the presence of a “y” shape palatine structure as in *Dsungaripterus*. In several other pterodactyloids such as *Anhanguera*^10,27,33,34^ (Anhangueridae, Fig. 3d, j, q), *Ludodactylus*^55^ (Ornithocheiridae, Fig. 3c) and probably also in *Caupedactylus*^38^ (Tapejaridae, Fig. 3h), also present a similar presence of a “y” shape palatine.

Regarding palatal openings, several pterodactyloid taxa show three main palatal openings positioned lateral to the choanae. Based on our study, the most anterior of these openings should be the maxillopalatine fenestra. This appears to be the case of the tapejarid *Tupuxuara*^14^, and archaeopterodactyloid ctenochasmatids *Gnathosaurus*^12^ and *Liaodactylus*^42^ (Figs. 3j, i, 4c).

Some other pterodactyloids present only two openings lateral to the choana, such as *Pteranodon*^31^ (Pteranodontidae), *Tropeognathus*^26^ and *Anhanguera*^27,56^ (Anhangueridae), *Nyctosaurus*^19^ (Nyctosauridae), and *Aurorazhdarcho*^57^ (Ctenochasmatidae or *Pterodactylus*^23^, Pterodactylidae). The most anterior one, as in other pterodactyloids, is the maxillopalatine fenestra, followed by the subtemporal fenestra (Figs. 3k–s, 4d, e). This suggests that they lack a postpalatine fenestra. The main reason for this is the possible lack of the development of a lateral process of the ectopterygoid, that in the other studied pterodactyloid separates postpalatine fenestra from the subtemporal fenestra (Fig. 6c). In some specimens of *Anhanguera* (SNSB-BSPG 1982 I 89), *Hongshanopterus*, *Gnathosaurus*, *Aurorazhdarcho*, the ectopterygoid shows a blunt incipient process directed into the subtemporal opening.

As observed in non-pterodactyloids, some palatal elements might also be reinterpreted in pterodactyloids. The palatine as presented in previous studies^12,23,26–28,31,38,43,48^ is here reinterpreted as the median maxilla process (Fig. 6c). Also the bone interpreted as the ectopterygoid in some taxa^28,31,35,38,42,43,48,58,59^ are here considered as being the palatines, which shows two rami as observed in the CT-scans of *Dsungaripterus*.

The diverse shape of the maxillopalatine fenestra observed in non-pteradactyoids and pterodactyloids (Figs. 2, 3,4) is influenced by several factors, including the shape and extension of the palatine process of the maxilla, the angle between the two rami of palatine, and the contact of the palatine lateral ramus with bony bar formed by maxilla and jugal. The angle between the two rami of the palatine varies from ~30° in *Dsungaripterus*, *Caupedactylus*, *Tupuxuara*, *Nyctosaurus gracilis*, and *Pteranodon*, ~45° in *Hongshanopterus*, *Aurorazhdarcho*, *Liaodactylus primus*, and *Gnathosaurus*, and 45°–60° in *Hamipterus* and *Anhanguera*.

In the region between the choanae and the interpterygoid fenestra, a pair of small openings are present and vary within pterodactyloids in size. For example, in *Dsungaripterus* these openings are small, forming a pterygoid foramen, while in *Anhanguera*, *Hamipterus*, and *Caupedactylus* they are large, forming a pterygoid fenestra (Figs. 3,4). There seems to be some variation in the sizes of these openings, that could be observed in some specimens of *Anhanguera*, being more developed in some (SNSB-BSPG 1982 I 89, MN 4805-V, BSP 1987 I 46, and SAO 16494) and smaller in others (Fig. 3, RGM 401 880, AMNH 25555, SNSB-BSPG 1982 I 90, and AMNH 22555).

Although an extensive comparison of the pterosaur palate with other diapsids is beyond the scope of this paper, there are a few comments and differences that can be highlighted. The most significant is the presence of a maxillopalatine fenestra, which is absent in other diapsids^58,60,61^ and might turn out to be a synapomorphy of Pterosauria. In crocodyliforms, the suborbital fenestra is anteriorly formed by the maxilla and the palatine, and posteriorly by the pterygoid and ectopterygoid, lacking any maxillopalatine fenestra^62^. In prolacertiformes (e.g., ref. ^63^) and lepidosauromopha^58,60^, the internal naris is positioned at the lateral side of the palate, followed by a postpalatine fenestra, and lacks any extra opening between them. But the postpalatine fenestra is similar to the one observed in pterosaurs, since it is formed anteriorly by the palatine and posteriorly by ectopterygoid.

To conclude, the new relation established here between the palatine, ectopterygoid, maxilla, and pterygoid suggest some reinterpretation of the main palatal openings (Fig. 6d). Although it has to be acknowledged that there is still much work on the pterosaur palate necessary to be able to establish stronger evolutionary trends on this portion of the pterosaur skull, six main general remarks can be presented.

1. In some non-pterodactyloids (e.g., *Rhamphorhynchus* and *Cacibupteryx*), the anterior process of the pterygoids is not as developed as it is in pterodactyloids (Fig. 6d).
2. The angle between the two rami of the palatine in some non-pterodactyloids are nearly 90°, in *Kunpengopterus* is 45°, and in pterodactyloids are <60° (e.g., ~30° in *Dsungaripterus*, ~60° in *Hamipterus*).
3. In pterodactyloids the jugal process of the maxilla extends posteriorly until the ectopterygoid, but not in the non-pterodactyloids (*Rhamphorhynchus* and *Cacibupteryx*).
4. The last tooth is posterior to the anterior margin of maxillopalatine fenestrae in non-pterodactyloids (e.g., *Dorygnathus*, *Cacibupteryx*, *Rhamphorhynchus*, and *Kunpengopterus*), but is anterior to the maxillopalatine fenestrae in toothed pterodactyloids (e.g., *Hongshanopterus*, *Anhanguera*, *Tropeognathus*, *Liaodactylus*, *Gnathosaurus*, *Aurorazhdarcho*, *Hamipterus*, and *Dsungaripterus*).
5. Pterodactyloids have a pair of pterygoid openings (foramen or fenestra) bordered by the lateral ramus of the palatine and the median process of pterygoid. This opening is present in the non-pterodactyloid *Kunpengopterus* and *Cacibupteryx*, but not in *Rhamphorhynchus*.
6. Along with the great variation in the sizes of the palatal openings such as the pterygoid foramen or fenestra, the posterior margin of the choanae moved posteriorly to the maxillopalatine fenestra in *Kunpengopterus* and pterodactyloids (Fig. 6d) and even posterior to the postpalatine fenestra in some pterodactyloids (e.g., *Caupedactylus*, *Tupuxuara*, and *Dsungaripterus*).

### Supplementary information

Description of Additional Supplementary Files

Supplementary Data 1

Supplementary Video 1

Reporting Summary

## Data Availability

The authors declare that the main data supporting the findings of this study are available within the article and its Supplementary Information file. Extra data were available from the corresponding author or the first author upon request.
