# Supplementary material for: New insights into pterosaur cranial anatomy: X-ray imaging reveals palatal structure and evolutionary trends
Source: Commun Biol. 2024 Apr 12;7:456. doi: 10.1038/s42003-024-06132-6 (PMC11014945; doi:10.1038/s42003-024-06132-6)
Supplement: Supplementary file 1 — Description of Additional Supplementary Files [file 42003_2024_6132_MOESM1_ESM.pdf]

Description of Additional Supplementary Files

File name: Supplementary Data 1
Description: 3D reconstruction of the palate of *Dsungaripterus weii* IVPP V 4063.

File name: Video 1
Description: The CT-rendered results of the palate of *Dsungaripterus weii* IVPP V 4063.
